# Electrostatically Induced Recruitment of Membrane Peptides into Clusters Requires Ligand Binding at Both Interfaces

**DOI:** 10.1371/journal.pone.0052839

**Published:** 2012-12-21

**Authors:** Yuri N. Antonenko, Andreas Horner, Peter Pohl

**Affiliations:** 1 Belozersky Institute of Physico-Chemical Biology, Moscow State University, Moscow, Russia; 2 Institut für Biophysik, Johannes Kepler Universität, Linz, Austria; Swiss Federal Institute of Technology Zurich, Switzerland

## Abstract

Protein recruitment to specific membrane locations may be governed or facilitated by electrostatic attraction, which originates from a multivalent ligand. Here we explored the energetics of a model system in which this simple electrostatic recruitment mechanism failed. That is, basic poly-L-lysine binding to one leaflet of a planar lipid bilayer did not recruit the triply-charged peptide (O-Pyromellitylgramicidin). Clustering was only observed in cases where PLL was bound to both channel ends. Clustering was indicated (i) by the decreased diffusional PLL mobility *D_PLL_* and (ii) by an increased lifetime *τ_PLL_* of the clustered channels. In contrast, if PLL was bound to only one leaflet, neither *D_PLL_* nor *τ_P_* changed. Simple calculations suggest that electrostatic repulsion of the unbound ends prevented neighboring OPg dimers from approaching each other. We believe that a similar mechanism may also operate in cell signaling and that it may e.g. contribute to the controversial results obtained for the ligand driven dimerization of G protein-coupled receptors.

## Introduction

The association of proteins with the surfaces of plasma membranes or intracellular membranes is tightly regulated. Membrane affinity may be solely provided by electrostatic attraction of amino acid residues, which concentrate in the tertiary structure to form a binding surface [1]. Alternatively, phospholipid binding domains may be engaged, including e.g. pleckstrin homology domains and Fab1 domains [2]. When these domains interact with membranes, it involves stereospecific recognition of membrane targets like diacylglycerol and phosphoinositides. Coincidentally, protein attraction to the membrane may be aided by electrostatic or hydrophobic protein-lipid or protein-protein interactions.

Simultaneous involvement of several detection mechanisms is believed to be responsible for the restricted, rather than uniform distribution of recruited proteins across intracellular membranes. For example, detection of both phosphoinositides and small monomeric GTPases directs the four-phosphate adaptor protein-1 to the *trans*-Golgi network [3]. While the benefit of cluster formation for signaling purposes is immediately evident, the affinity requirements for protein and lipid recruitment into these clusters are less clear [2].

For example, it takes both lipidation and electrostatic lipid protein interactions to target the polybasic myristoylated alanine-rich PKC substrate peptide (MARCKS) [4] at the membrane. Once anchored to the membrane, MARCKS laterally recruits negatively charged phospholipids. A generalization of this phenomenon suggests that any basic peptide may recruit multivalent membrane anions into clusters [5]. However, when exchanging PiP_2_ for the triply charged gramicidin peptide o-pyromellitylgramicidin (OPg), no clusters were observed, i.e. unilateral binding of the basic poly-L-lysine (PLL) fails to recruit OPg [6].

OPg forms ion-conductive dimers due to C-termini interactions at the membrane midplane where *M* and *M_2_* denote the monomer and dimer, respectively. 
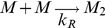
(1)


The ratio K of the respective association and dissociation constants, k_R_ and k_D_, is equal to the ratio of the equilibrium surface concentrations A and AA (both in units of mol cm^−2^) of M and M_2_, respectively:
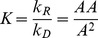
(2)


PLL's failure to induce the formation of conducting OPg dimers cannot be explained by the lack of a lipid anchor. PLL and OPg possess different mobilities and they rapidly exchange binding partners only in case of unilateral PLL binding. However, the lipid anchor is still absent when bilateral PLL binding is allowed. And yet in this case, measurements of current flow through OPg channels revealed cluster formation [7].

**Figure 1:**
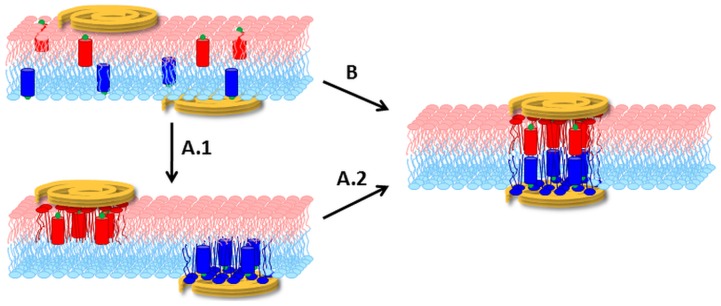
Genesis of a transmembrane cluster. We distinguish between two hypotheses: (i) Clustering occurs independently in the two leaflets (**A.1**). The clusters subsequently register (**A.2**); (ii) The transmembrane cluster forms as entities that are immediately in register across both leaflets upon their formation (**B**).

The goal of the present paper is to clarify the mechanism and energetics of this simple OPg – PLL model system. Thus, we aspire (i) to distinguish whether the transmembrane cluster emerges by registration of half-clusters in the individual leaflets or by recruitment of entities that register across both leaflets immediately upon cluster formation and (ii) to understand why PLL binding to both channel entry and channel exit is required for cluster formation (Fig. 1).

## Materials and Methods

### Planar membranes

Vertical planar bilayer lipid membranes were formed by painting diphytanoyl phosphatidylcholine (DPhPC) solution in decane (20 mg/ml) over an aperture (500 µm in diameter) in a diaphragm separating two aqueous solutions. Horizontal planar bilayers were folded from DPhPC monolayers to cover the aperture (100 µm in diameter). The apertures were pretreated with 2% DPhPC in n-decane or with 0.5% hexadecane in hexane, respectively. The volumes of the lower and upper chambers were 3 and 0.5 ml, respectively. We observed formation of planar membranes (i) optically, either through a front window or through a cover glass in the bottom of the lower chamber [8] and (ii) electrically, via the determination of membrane capacitance. Electrical current was measured by means of a picoamperemeter (Keithley Instruments or VA-10 amplifier, npi, Tamm, Germany).

Most of the experiments were performed in a solution containing 25 mM KCl, 10 mM HEPES, and 0.1 mM EDTA buffered at pH 7. The ethanolic stock solution (0.015 mg/ml OPg) was mixed with the DPhPC/decane membrane-forming solution or OPg (a generous gift from N. S. Melik-Nubarov, Moscow State University, Department of Chemistry) was added from a stock solution of 2 mg/ml to the DPhPC/hexane monolayer which was formed on top of the aqueous solution.

Cy3 conjugated gramicidin A (gCy3, a generous gift provided by V. Borisenko and G.A.Woolley, University of Toronto, Canada) was prepared as previously described [9]. Cy3 attachment did not affect channel activity [9].

PLL (PLL, Sigma, Vienna, Austria) was added to one or both compartments of the cell as stated. In some experiments, PLL was labeled with Atto633. PLL, HBr (average molecular weight 24,000, 115 Lysines, Sigma), was dissolved in double-destilled water (pH 9, 2 mM) and mixed with equal amounts of peroxide-free dioxan. 1 mM Atto633-NHS (Atto Tec, Siegen, Germany) were added and incubated in the dark for 2 h under Argon. The Atto633 labeled PLL_115_ was subsequently lyophilized, redissolved in bi-destilled water and stored at 4°C. Experiments were carried out at room temperature (21–23°C).

### Fluorescence correlation spectroscopy (FCS) measurements

The surface diffusion coefficients of gCy3 and PLL(Atto633) were measured by fluorescence correlation spectroscopy (ConfoCor 3 attached to the laser scanning microscope LSM510, Carl Zeiss, Jena, Germany). The dyes were excited at 561 and 633 nm. We calibrated the confocal volume by measuring the residence time *τ_R_* of rhodamine 6G in solution. Based on a diffusion coefficient of 426 µm^2^ s^−1^ [10], we obtained confocal plane radii *ω* of 0.22 μm and 0.25 μm for the two different lasers.

Autocorrelation functions *G(τ)* of the fluorescence temporal signal from PLL-Atto633 were fitted to the two-dimensional equation
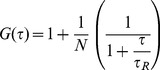
(3)where *N* is the number of particles. We performed so-called Z-scans to exactly position the horizontal membrane in the focus. We gradually changed the vertical position of the laser focus relative to the phospholipid surface plane [11]. Recordings made right in the focus were used to estimate *D_PLL_*. The absolute value of *D_PLL_* was determined with an accuracy of about 20% [12]. Only relative changes in *D_PLL_* are important for the scope of the current work and these were determined with much higher precision.

### Evaluation of changes in the dissociation kinetics of the OPg dimers induced by cluster formation

The association of two OPg monomers from two different leaflets resulted in the formation of a transmembrane pore. These cation-conducting dimers of labeled gramicidin derivatives are comparable to the wild type channel, both are stabilized in their head-to-head association by six hydrogen bonds [13]. The lifetime of these OPg dimers depends on the force exerted to decrease the membrane thickness to the size of the dimer [14]. If the number of open channels is small, it can be monitored by single channel recordings. If hundreds or thousands of open channels are reconstituted, the decay time of the transmembrane current subsequent to sudden removal of functional monomers can instead be used [15].

The monomer-dimer equilibrium instantaneously shifts upon photodynamic monomer inactivation [16], . The photosensitizer, aluminum trisulfophthalocyanine (AlPcS_3_, Porphyrin Products, Logan, UT), therefore adsorbed to the membrane and generated singlet oxygen ^1^O_2_ by a flash of light. The ^1^O_2_ diffusion span within the membrane [18] is sufficient to target tryptophan residues of gramicidin monomers [19]. Since both the duration of the flash and the lifetime of ^1^O_2_ [20] are at least two orders of magnitude smaller than the characteristic decay time (*τ_P_* ≈ 0.2 s) of the membrane current through the OPg dimers [7], they can generally be neglected. *τ_P_* is obtained from a single exponential fit of the equation:
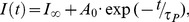
(4)to the current trace recorded after a flash of light. *I_∞_* denotes the final membrane current. The addition of appropriate concentrations of polymers leads to two-exponential kinetics:



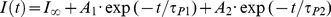
(5)It can be shown that in the case of a mixture of two exponentials, single exponential fit should reveal *τ_P_*:
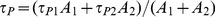
(6)


We calculated *τ_P_* from an exponential fit (Eq. 5, Eq. 6) or we obtained *τ_P_* by fitting the data with a single-exponential function (Eq. 4).

AlPcS_3_ was added to the bathing solution at the trans-side. The flash lamp was attached to the cis compartment. The current *I* was measured under voltage-clamp conditions by a current amplifier (model 428, Keithley Instruments), digitized by using a LabPC 1200 (National Instruments, Austin, TX) and analyzed using a personal computer with the help of WinWCP Strathclyde Electrophysiology Software designed by J. Dempster (University of Strathclyde, UK). Ag-AgCl electrodes were placed directly into the cell and a voltage of 30 mV was applied to the lipid bilayer. The value of the current was about 1 µA on average which corresponded to 7.5×10^6^ conducting channels in the bilayer. Planar lipid bilayers were illuminated by single flashes produced by a xenon lamp with flash energy of about 400 mJ/cm^2^ and flash duration <2 ms. A glass filter was placed in front of the flash lamp to cut off light with wavelengths <500 nm. To avoid electrical artifacts, the electrodes were covered by black plastic tubes.

## Results

**Figure 2:**
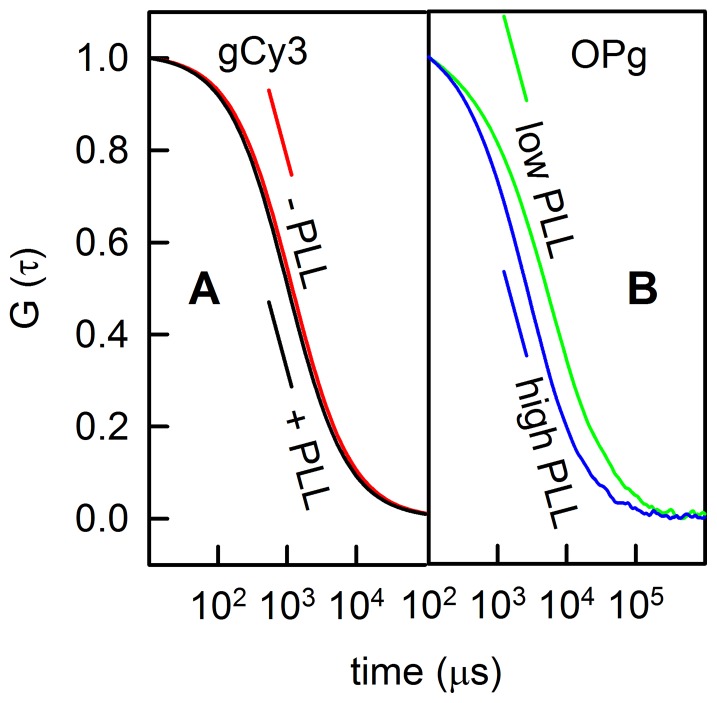
PLL (115 residues) binding to free-standing planar membranes. (**A**) Representative autocorrelation curves obtained for gCy3-doped planar lipid bilayers prior to (red line) and subsequent to (black line) the addition of PLL to both sides of the membrane. (**B**) FCS autocorrelation functions for labeled PLL (Atto633) added to both sides of OPg-doped planar lipid bilayers at a concentration of ∼ 0.7 µM (green line) and ∼ 500 µM (blue line) per lysine monomer. Only triply charged OPg, not singly charged gCy3 interacted with PLL strong enough so that a decrease in mobility became measureable (green line). The autocorrelation function indicates the absence of clusters (blue line). It is thus similar to autocorrelation functions for PLL concentrations (per lysine residues) in the nM range and >100 µM. The membranes in **A** were painted from a 50∶50 (V:V) % mixture of lipid (20 mg DPhPC per ml decane) and gCy3 (0.001 mg per ml ethanol). The membranes in **B** were folded from monolayers containing OPg (0.5 mg OPg and 20 mg DPhPC per ml hexane). The bathing solution was 25 mM KCl, 10 mM HEPES, 0.1 mM EDTA, pH 7.



Free-standing planar bilayers doped with gCy3 were placed into the focus of the laser scanning microscope. Diffusion of the dye into and out of the focus resulted in fluorescence intensity fluctuations. Calculation of the corresponding autocorrelation function (Fig. 2A) allowed determination of gCy3 residence time *τ_R_* in the focus. Computation of the membrane diffusion coefficient D_M_ according to Eq. (7):

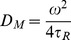
(7)resulted in a value of 8.9±0.8 µm^2^/s. D_M_ is two to three times larger than that measured by single particle tracking under comparable conditions [21]. However, it was close to the *D_M_* of lipids which was determined to be 8.1±0.4 µm^2^/s [12], [22]. The similarity between *D_M_* of lipids and peptides with one membrane helix is in line with measurements of fluorescence recovery after photobleaching [23]. Addition of PLL in any concentration to one or both sides of the membrane did not alter D_M_. This result nicely agrees with the observation made by Ghambhir et al. [24] that recruitment into clusters requires the number *z* of charges per molecule to be ≥2. To test this hypothesis we substituted gCy3 for OPg (z = 3).

**Figure 3:**
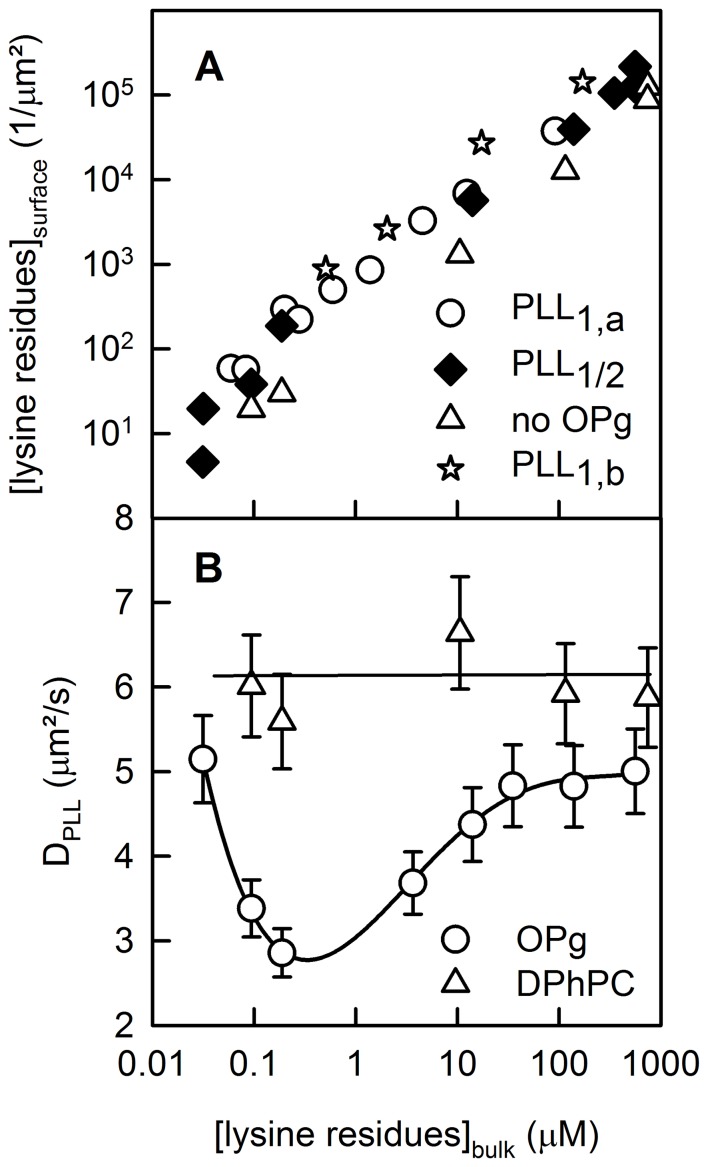
Membrane bound PLL. PLL surface concentration *PLL_surface_* (**A**) and PLL diffusion coefficient *D_PLL_* (**B**) as functions of the bulk concentration of lysine residues. Due to its small contribution to the overall surface potential (∼300 charges/µm^2^), OPg had a small effect on PLL surface concentration. Unilateral and bilateral additions are marked with the indices 1 and 2, respectively. The subscripts a and b indicate the different leaflets (A). Both *PLL_surface_* and *D_PLL_* were determined from FCS autocorrelation functions. *D_PLL_* is equal to the ratio *ω^2^/4τ_R_*, where *ω* and *τ_R_* are the radius of the confocal plane and the mean residence time of PLL-Atto633 in the focus, respectively. The ratio of unlabeled to labeled PLL increased from zero to ∼1000 with increasing PLL concentration. In the absence of OPg, *D_PLL_* was insensitive to the amount of PLL added (curve labeled DPhPC). The same observation was made when PLL was allowed to adsorb to one leaflet only. *D_PLL_* decreased in a narrow PLL concentration interval (B), only when present at both sides of an OPg-containing membrane. The membranes were folded from DPhPC monolayers (20 mg per ml hexane). OPg was added at a concentration to form ∼ 50 dimers per µm^2^ which was checked by conductivity measurements prior to the addition of PLL. The single channel conductance was assumed to be equal to 4 pS. The bathing solution was 25 mM KCl, 10 mM HEPES, 0.1 mM EDTA, pH 7.



We measured a PLL diffusion coefficient *D_PLL_* of 6±1 µm^2^/s (Fig. 3B) upon unilateral PLL adsorption to the surface of free-standing planar bilayers made of DPhPC (Fig. 3A). We calculated *D_PLL_* similar to *D_M_* (Eq. 7). Increasing the PLL concentration from 10^−7^ to 10^−4^ M had no effect on *D_PLL_*. Even reconstitution of OPg in a concentration of up to ∼ 50 dimers per µm^2^ did not alter *D_PLL_* (Fig. 3B). Since OPg was not labeled, we determined its surface density as the ratio of the transmembrane conductivity to the single channel conductivity and the membrane area. Eq. (2) revealed that *A* matched *AA* for K = 1.2×10^14^ cm^2^ mol^−1^ [25], i.e. the monomer was present at a surface density ∼ 50 µm^−2^. Taking into account that every OPg bears three negative charges, we obtained a density of about 300 charges per µm^2^. According to the Gouy-Chapman theory, this charge density *σ* corresponds to a surface potential *ψ_0_* of:

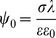
(8)Assuming that the permittivity *ε* at the membrane surface is equal to 10, we find that the Debye length *λ* is equal to 0.68 nm, which results *ψ_0_* ≈ −0.37 mV. Thus, OPg makes a negligible contribution to membrane surface potential, because membranes formed from neutral lipids have a surface potential of around −6 mV [26], [27]. As a consequence, OPg has only a very modest effect on PLL adsorption to the membrane (Fig. 3A).

Despite the small *ψ_0_, D_PLL_* dropped significantly when PLL was added to both sides of the membrane. The effect depended on PLL concentration. Above a threshold bulk concentration of about 10^−7^ M (per lysine monomer), it became apparent that *D_PLL_* reached its minimum at about 10^−6^ M (Fig. 2B, green curve) and returned to the initial value for bulk concentrations >10^−5^ M (Fig. 3B). The peak of cluster formation was observed at a polymer density of 5/µm^2^ (Fig. 3A). If we take the drop in *D_PLL_* as an indicator for transmembrane cluster formation, several OPg dimers must have been embedded between two PLL molecules.

The hydrophobic thickness of lipids and gramicidin differ by *l* ∼ 0.3 nm [28]. As a result, a line tension around an isolated OPg dimer or around a cluster with several OPg dimers must exist [14]. We extrapolated *σ* ∼ 5 pN from tension values measured as a function of hydrophobic mismatch between lipid clusters [29]. Assuming that the cluster is circular to minimize the energy per boundary length, we calculated the lipid deformation energy ΔG which is spent when an OPg dimer forms, i.e. the channel opens as:
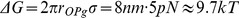
(9)where *r_OPg_* is the radius of an OPg dimer. It is calculated assuming an effective cross-sectional area of OPg, A_OPg_ ∼ 5 nm^2^, consisting of 2.5 nm^2^ for OPg itself [30] and 0.63 nm^2^ for each of the four bound lipids [31]. The result of the oversimplified Eq. (9) is in good agreement with calculations of ΔG from a so-called phenomenological spring constant *H_B_* [32]. For bilayers made from dioleoylphosphatidylcholine in decane, *H_B_* is equal to 56 kJ mol^−1^ nm^−2^ [33] so that ΔG  =  H_B_ × (2×*l*)^2^ ≈ 8.2 kT.

For a cluster of *n* OPg molecules Eq (9) transforms into:
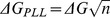
(10)where *Δ*G_PLL_ is the lipid deformation energy per cluster. According to Eq. 10, the lipid deformation energy *ΔΔG* per OPg dimer in the cluster is smaller than *Δ*G:



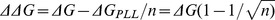
(11)Eq. 11 indicates a reduction of strain on the six hydrogen bonds between the monomers of an individual OPg dimer. Hence, OPg dimer lifetime increases in a cluster [34]–[36].

**Figure 4:**
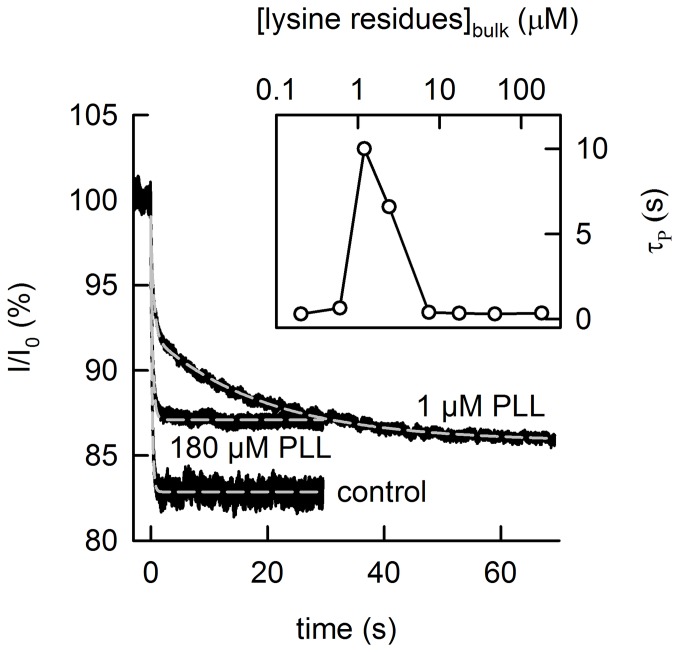
PLL binding increases the lifetime *τ_P_* of OPg dimers. The sudden decrease of the OPg monomer fraction by photorelease of singlet oxygen (from 1 µM AlPcS_3_) results in an exponential decay of the OPg-mediated current *I* through planar bilayers. The time constant of the decay *1/τ_P_* depends on the PLL concentration in the bathing medium. *τ_P_* is obtained from a single exponential fit of Eq. (4) to the data (dashed gray lines). *τ_P_* is equal to 0.23 s (concentration per lysine residue  = 180 µM PLL) and 0.34 s in the absence of PLL (control), respectively. Double-exponential fitting (dashed gray line) is required (according to: I = I_0_+A_1_exp(−t/τ_P1_)+A_2_exp(−t/τ_P2_)) in the presence of 1 µM PLL at both sides of the membrane. The best fit is attained with the parameters: A_1_ = 6.7%, τ_P1_ = 0.53 s, A_2_ = 6.2%, τ_P2_ = 19.5 s. The initial value of the current *I_0_* was ∼ 1 µA. Insert: the dependence of the averaged *τ*_P_ (compare Eqn. 6) on bulk PLL concentration.



To estimate *n*, we probed OPg dimer dissociation kinetics by photoinactivation of OPg monomers. The transmembrane current (which was initially about 1 μA) decayed with time constant *τ_P_*, which was roughly equal to OPg dimer lifetime of 0.2s. *τ*_P_ increased about a hundredfold to the new value τ_PLL_ ≈ 20s, when PLL bulk concentration reached ∼ 1 μM PLL (per lysine monomer). It returned to control values at both much lower and much higher PLL concentrations (Fig. 4). *τ_PLL_* and *τ_P_* relate to *ΔΔG*:

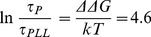
(12)Inserting the values for *ΔΔG* and *ΔG* into Eq. 11, we calculate that *n* = 4 OPg dimers are bunched into one cluster.

In contrast to the case of bilateral PLL presence discussed above, the addition of PLL to only one side of the membrane did not result in the deceleration of the photo-inactivation kinetics, indicating that clusters do not form isolated in one leaflet. Similar results have previously been reported for experiments carried out at higher ionic strength [37]. The observation agrees well with *D_PLL_* measurements which also indicated the absence of clusters.

**Figure 5:**
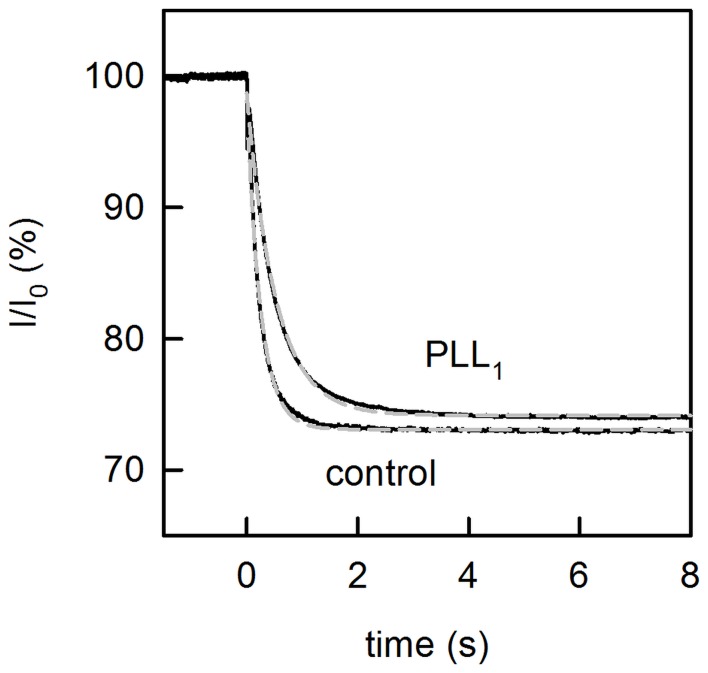
Lack of cluster formation upon unilateral PLL addition. Unilateral PLL addition at a concentration of 2.5 µM per lysine monomer, only has a minor effect on the lifetime *τ_P_* of OPg dimers even at 1 M KCl on the opposite side. The sudden decrease of the OPg monomer fraction by photorelease of singlet oxygen (from 1 µM AlPcS_3_) results in an exponential decay of the OPg-mediated current *I* through planar bilayers. The time constant *τ_P_* of the decay is obtained from a single exponential fit of Eq. (4) to the data (dashed gray lines). *τ_P_* is equal to 0.25 s in the absence of PLL (control) and 0.52 s in the presence of PLL, respectively. The initial value of the current *I_0_* was ∼ 1 µA.

To test the hypothesis that clustering was opposed by the electrostatic repulsion of the unbound channel ends, we increased the ionic strength in the compartment lacking PLL. We observed only a very modest increase of *τ_p_* from 0.25 s to 0.5 s (Fig. 5), which indicated the persistent lack of OPg clusters.

## Discussion

**Figure 6:**
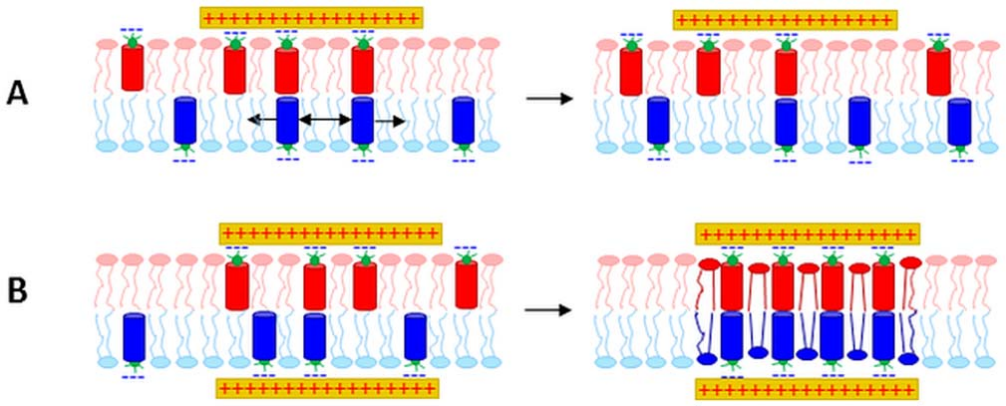
Scheme of cluster formation as entities that are immediately in register across both leaflets upon their formation. (**A**) Pure electrostatic interactions between OPg and unilaterally adsorbed PLL does not lead to cluster formation. PLL mobility and OPg dimer lifetime are unaltered. (**B**) Formation of an OPg dimer which is simultaneously bound by two opposing PLL molecules leads to cluster formation. The process is driven by electrostatic attraction leading to a local increase in OPg concentration and by the energetically favorable formation of additional OPg dimers.

There are two alternative ways that a membrane-spanning cluster may form: (i) The constituents are first separately recruited in each individual leaflet into half-clusters, which in a second step form a transmembrane cluster or (ii) components from different leaflets first align forming a nucleus, which in a second step develops into a cluster by the concerted recruitment of more constituents from both leaflets. PLL's inability to cluster OPg monomers excludes mechanism (i), while recruitment of multiple OPg dimers to bilaterally bound PLL confirmed mechanism (ii) (Fig. 6).

The OPg and PLL concentrations at which cluster concentration reaches a maximum, translate into average distances of about ∼ 447 nm and ∼ 100 nm between neighboring PLL molecules and neighboring OPg molecules, respectively. Even a fully extended PLL molecule with a length of only ∼ 45 nm does not bridge this distance. However, the molecule diffuses so fast (D_PLL_ = 5 µm^2^/s), that within the 0.2 s lifetime of an OPg dimer, it crosses a distance of 
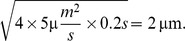
 As a consequence, one PLL molecule encounters about 2 µm/0.1 µm  = 20 OPg molecules during 0.2 s. On average about 10 of these 20 OPg molecules are present as dimers. Thus, in a statistical ensemble there should be a significant population of PLL molecules which are bound to several OPg monomers or dimers at the same time. The electrostatic attraction between OPg and PLL acts to increase this population.

Once an OPg monomer binds to an already existing PLL-OPg-dimer complex, the increased surface density augments the likelihood of dimerization (compare Eq. 2). The fact that 2 OPg molecules cannot be more than 45 nm apart translates into a minimal OPg surface density of 1.3×10^5^/µm^2^, which shifts the minimal equilibrium dimer to monomer ratio to 0.98∶0.02. Because OPg clustering reduces the energy *ΔG* which is incurred by bilayer thinning at the peptide OPg interface to *ΔΔG*, K increases a hundredfold. This conclusion is based on the observation that *τ_PLL_ ∼* 100×*τ_P_*. The combined effect of increased OPg surface density and the augmented *K* value of 1.2×10^16^ cm^2^/mol are equivalent to a shift in the dimer to monomer equilibrium from 0.5∶0.5 (Eq. 2) to 0.998∶0.002.

From the difference in *ΔΔG* and *ΔG*, we estimated a cluster size of four OPg dimers. This corresponds very well to the area ∼ 22 nm^2^ of a condensed PLL molecule (115 residues) on charged planar bilayers [22]. Our current FCS measurements also agree with the anticipated cluster size. Cluster formation implies that OPg and PLL diffuse as one entity. That is, D_M_ decreased threefold from its initial value of 8.9×10^−8^ cm^2^ s^−1^ to 2.8×10^−8^ cm^2^ s^−1^. The first number stems from the assumption that *D_M_* of gCy3 and of OPg are similar. The second value reflects the situation in which most of the PLL molecules are part of a cluster (minimum in Fig. 3B). Such a change in *D_M_* indicates a ninefold increase in molecular weight, i.e. the radius of the diffusing entity increased from that of one OPg dimer (0.89 nm) to that of a tetramer of OPg dimers with 16 tightly bound lipids (2.6 nm). This calculation assumes that the 8 lipids bound to the dimer must be interchangeable, because they do not contribute to *D_M_*, while the 32 lipids are locked in the cluster which is sandwiched between two PLL molecules (Fig. 6B). The inability of the clustered lipids to exchange with the surrounding lipids has been previously observed and has been attributed to the line tension around the cluster [22]. Cluster size estimates are further based on the inverse proportionality of *D_M_* and the molecular radius [23]. The logarithmic dependence of the membrane diffusion coefficient on cluster size as described by the Saffman-Delbrück formalism [38] should be used for larger clusters (radius >3 nm).

When PLL unilaterally binds, the above analysis does not explain the lack of clusters. Insight is expected from a closer look at the unfavorable total energy balance:
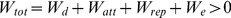
(13)where *W_d_*, *W_att_*, *W_rep_* and *W_e_* denote the dimerization energy, the attractive electrostatic energy, the repulsive energy and the entropy-induced amount of energy, respectively.

To calculate the dimerization energy *W_d_*, we take into account that the concentrations of OPg monomers and dimers are equal to each other at the highest cluster abundance (Eq. 2). The assembly of four OPg molecules in the small spot of one PLL molecule must consequently be accompanied by the formation of two new OPg dimers, which both contribute to E_D_. The dissociation energy of a gramicidin dimer *E_D_* is about −28.7 RT [25], where *R* is the gas constant and *T* the absolute temperature. Dimerization therefore contributes *W_d_ = 2 x E_D_  = −57.4 RT* to cluster formation. *ΔG_PLL_* is equal to 2× *ΔG* (Eq. 10), so that neither *ΔG_PLL_* nor *ΔG* appear in Eq. 13.

The attractive electrostatic energy *W_att_* is fourfold larger than the electrostatic energy *W_el_* between one OPg and PLL [39]. For the assessment of *W_el_* we use the textbook equation:
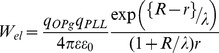
(14)where *R = 0.9 nm, r = 0.9 nm*, and *q_OPg_  = −3e* are the OPg radius, the average distance between the interacting charges, and the OPg charge, respectively. *e* is the elementary charge. We estimate *ε* to be ∼ 10 on the membrane surface. Because PLL is treated as a point charge in Eq. (14), we have to derive an effective charge *q_PLL_*. *q_PLL_* accounts for (i) the size of the polymer and (ii) the steric restraints which arise from its interactions with other (OPg) molecules. For an assessment of *q_PLL_* we used data from a previous publication [37] in which τ_PLL,_ induced by the 60 residues large PLL (PLL_60_), was a function of the ionic strength of the bathing solution. Krylov et al. [37] observed the largest *τ_P_* at PLL_60_ bulk concentrations (in monomer units) *c_L_*  = 10^−6^ M, 10^−5^ M, and 3×10^−5^ M in 50, 100 or 150 mM KCl, respectively. Assuming that these concentrations are proportional to the apparent dissociation constant *K_D,app_* of the PLL-OPg complex at a given ion concentrations, we can write:
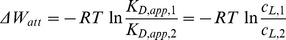
(15)for any pair of *c_L_*. That is, increasing the KCl concentration from 50 to 100 or to 150 mM KCl changed the attractive electrostatic energy, *W_att_*, by 2.3 RT or 3.4 RT, respectively. There must have only been three OPg dimers per cluster in these experiments, because the square root of the number of residues is proportional to the area of the cluster [40]. That is, *ΔW_att_* must be divided by six (the number of pyromellityl groups per dimer) to obtain the decrement in *W_el_* introduced by the increment in KCl concentration. Inserting *q_PLL_*  =  *0.5e* into Eq. (13) satisfies this requirement. It is safe to assume that *q_PLL_* does not change with an increase in the number of residues, because the additional residues are distant from OPg. For 25 mM KCl we calculate *W_el_* ≈ *-4.1 RT* or *W_att_*  =  *-16.4 RT*.

At the PLL free leaflet, the unbound pyromellityl groups repel each other with *W_rep_*. Substituting *q_PLL_* for *q_OPg_* and assuming *r = 0.9 nm* allows utilization of Eq. (13) for calculation of the repulsive electrostatic energy *W_el_ ≈ 25 RT* between any pair of OPg molecules. In a cluster of four OPg dimers, there are six such pairs so that the total repulsive energy *W_rep_* amounts to 150 RT.

The entropy-induced amount of energy *W_e_* depends on the probability *p* that 4 OPg monomers (two from each monolayer) and 2 OPg dimers simultaneously hit the 22 nm^2^ large spot, which is occupied by one condensed PLL molecule. There is only one such spot per 200,000 nm^2^ at the highest cluster abundance. As it is tenfold more abundant, an individual OPg monomer or an individual OPg dimer encounters one PLL molecule with a probability of *p_m/d_* ≈ 1.1×10^−3^. Thus, a rough estimate for p ≈ (p_m/d_)^6^ results in 1.1×10^−18^. In turn, we estimate *W_e_* to be equal to – *RT ln p ≈ 41 RT*.

Now we are able to calculate the total energy balance (Eq. 13):
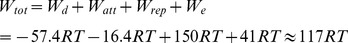
(16)


It confirms our hypothesis: the repulsion between the charged OPg groups dominates. Cluster formation is only energetically favorable in the absence of *W_rep_*. To further validate the result, we decreased *W_rep_* by increasing the ionic strength (Fig. 5). In a solution of 1 M KCl, *W_el_* is equal to 6.1 RT between any pair of OPg molecules, (Eq. 14), which translates into *W_rep_ ≈ 37 RT*. Thus, *W_tot_* amounts to ∼ 4.2 RT, suggesting that cluster formation remained unfavorable. This conclusion is in perfect agreement with the experiment (Fig. 5).

Eq. (16) also explains the dependence of cluster concentration on PLL and OPg concentrations in case of bilateral PLL addition, at least on a qualitative level. A tenfold increase in PLL concentration shifts the interfacial OPg/PLL ratio to 2∶1, causing a tremendous increase in entropy-induced amounts of energy for the simultaneous binding of 4 OPgs to one PLL. A tenfold increase in the OPg concentration shifts the OPg dimer: monomer to 13∶1, which vanquishes W_d_. W_att_ cannot drive cluster formation on its own, because it is smaller than W_e_.

In summary, the PLL-induced buildup of OPg clusters isolated in one leaflet is opposed by electrostatic repulsion from the opposite leaflet. We believe that the insight gained by studying this model system may be helpful for understanding the much more complex aggregation of receptors in the cellular plasma membrane. For example, it may shed light onto the highly controversial issue [41] of ligand-induced dimerization of certain G-protein-coupled receptors (GPCRs) [42]. The critical extracellular GPCR ligand binding sites and the intracellular docking sites for G-protein both contain charges. These charges are conserved throughout the G protein-coupled receptor family. For example, negative charges are excluded in peptide-GPCRs, whereas positive charges are excluded from the critical extracellular locus in amine-GPCRs [43]. Certain charged residues of the cytoplasmic loops are likewise crucial for C-protein coupling, as was e.g. shown for the second inner loop of the muscarinic receptor [44]. Our study suggests that charge shielding by both ligands and G-proteins may in part regulate the extent to which some of the GPCRs form dimers.

## References

[1] Horner A, Goetz F, Tampe R, Klussmann E, Pohl P (2012) Mechanism for targeting the A-kinase anchoring protein AKAP18δ to the membrane. J Biol Chem. in press. 10.1074/jbc.M112.414946 PMC3522251 23095754

[2] Lemmon MA (2008) Membrane recognition by phospholipid-binding domains. Nat Rev Mol Cell Biol 9: 99–111. 18216767 10.1038/nrm2328

[3] Carlton JG, Cullen PJ (2005) Coincidence detection in phosphoinositide signaling. Trends Cell Biol 15: 540–547. 16139503 10.1016/j.tcb.2005.08.005 PMC1904488

[4] McLaughlin S, Murray D (2005) Plasma membrane phosphoinositide organization by protein electrostatics. Nature 438: 605–611. 16319880 10.1038/nature04398

[5] Golebiewska U, Gambhir A, Hangyas-Mihalyne G, Zaitseva I, Radler J, et al (2006) Membrane-bound basic peptides sequester multivalent (PIP2), but not monovalent (PS), acidic lipids. Biophys J 91: 588–599. 16648167 10.1529/biophysj.106.081562 PMC1483118

[6] Krylov AV, Rokitskaya TI, Kotova EA, Yaroslavov AA, Antonenko YN (2002) Kinetically different populations of O-pyromellityl-gramicidin channels induced by poly-L-lysines in lipid bilayers. J Membr Biol 189: 119–130. 12235487 10.1007/s00232-002-1007-7

[7] Krylov AV, Antonenko YN, Kotova EA, Rokitskaya TI, Yaroslavov AA (1998) Polylysine Decelerates Channel Kinetics of Negatively Charged Gramicidin as Shown by Sensitized Photoinactivation. FEBS Lett 440: 235–238. 9862462 10.1016/s0014-5793(98)01462-8

[8] Serowy S, Saparov SM, Antonenko YN, Kozlovsky W, Hagen V, et al (2003) Structural proton diffusion along lipid bilayers. Biophys J 84: 1031–1037. 12547784 10.1016/S0006-3495(03)74919-4 PMC1302680

[9] Lougheed T, Borisenko V, Hand CE, Woolley GA (2001) Fluorescent gramicidin derivatives for single-molecule fluorescence and ion channel measurements. Bioconjug Chem 12: 594–602. 11459465 10.1021/bc010006t

[10] Petrasek Z, Schwille P (2008) Precise measurement of diffusion coefficients using scanning fluorescence correlation spectroscopy. Biophys J 94: 1437–1448. 17933881 10.1529/biophysj.107.108811 PMC2212689

[11] Benda A, Benes M, Marecek V, Lhotsky A, Hermens WT, et al (2003) How to determine diffusion coefficients in planar phospholipid systems by confocal fluorescence correlation spectroscopy. Langmuir 19: 4120–4126.

[12] Przybylo M, Sykora J, Humpolickova J, Benda A, Zan A, et al (2006) Lipid Diffusion in Giant Unilamellar Vesicles Is More than 2 Times Faster than in Supported Phospholipid Bilayers under Identical Conditions. Langmuir 22: 9096–9099. 17042516 10.1021/la061934p

[13] Andersen OS, Apell HJ, Bamberg E, Busath DD, Koeppe RE, et al (1999) Gramicidin channel controversy – the structure in a lipid environment. Nat Struct Biol 6: 609. 10404209 10.1038/10648

[14] Goulian M, Mesquita ON, Fygenson DK, Nielsen C, Andersen OS, et al (1998) Gramicidin channel kinetics under tension. Biophysical Journal 74: 328–337. 9449333 10.1016/S0006-3495(98)77790-2 PMC1299385

[15] Bamberg E, Lauger P (1973) Channel formation kinetics of gramicidin A in lipid bilayer membranes. J Membr Biol 11: 177–194. 4131309 10.1007/BF01869820

[16] Rokitskaya TI, Antonenko YN, Kotova EA (1996) Photodynamic inactivation of gramicidin channels:a flash-photolysis study. Biochim Biophys Acta 1275: 221–226. 8695636 10.1016/0005-2728(96)00025-4

[17] Rokitskaya TI, Block M, Antonenko YN, Kotova EA, Pohl P (2000) Photosensitizer binding to lipid bilayers as a precondition for the photoinactivation of membrane channels. Biophys J 78: 2572–2580. 10777753 10.1016/S0006-3495(00)76801-9 PMC1300846

[18] Sokolov VS, Pohl P (2009) Membrane transport of singlet oxygen monitored by dipole potential measurements. Biophys J 96: 77–85. 18931253 10.1529/biophysj.108.135145 PMC2710020

[19] Kunz L, Zeidler U, Haegele K, Przybylski M, Stark G (1995) Photodynamic and radiolytic inactivation of ion channels formed by gramicidin A: oxidation and fragmentation. Biochemistry 34: 11895–11903. 7547925 10.1021/bi00037a030

[20] Ehrenberg B, Anderson JL, Foote CS (1998) Kinetics and yield of singlet oxygen photosensitized by hypericin in organic and biological media. Photochem Photobiol 68: 135–140. 9723207

[21] Borisenko V, Lougheed T, Hesse J, Fureder-Kitzmuller E, Fertig N, et al (2003) Simultaneous optical and electrical recording of single gramicidin channels. Biophys J 84: 612–622. 12524314 10.1016/S0006-3495(03)74881-4 PMC1302642

[22] Horner A, Antonenko YN, Pohl P (2009) Coupled diffusion of peripherally bound peptides along the outer and inner membrane leaflets. Biophys J 96: 2689–2695. 19348751 10.1016/j.bpj.2008.12.3931 PMC2711291

[23] Gambin Y, Lopez-Esparza R, Reffay M, Sierecki E, Gov NS, et al (2006) Lateral mobility of proteins in liquid membranes revisited. Proc Natl Acad Sci U S A 103: 2098–2102. 16461891 10.1073/pnas.0511026103 PMC1413751

[24] Gambhir A, Hangyas-Mihalyne G, Zaitseva I, Cafiso DS, Wang J, et al (2004) Electrostatic sequestration of PIP2 on phospholipid membranes by basic/aromatic regions of proteins. Biophys J 86: 2188–2207. 15041659 10.1016/S0006-3495(04)74278-2 PMC1304070

[25] Bamberg E, Lauger P (1974) Temperature-dependent properties of gramicidin A channels. Biochim Biophys Acta 367: 127–133. 4138938 10.1016/0005-2736(74)90037-6

[26] Cevc G (1990) Membrane electrostatics. Biochim Biophys Acta 1031: 311–382. 2223819 10.1016/0304-4157(90)90015-5

[27] Missner A, Horner A, Pohl P (2008) Cholesterol's decoupling effect on membrane partitioning and permeability revisited: Is there anything beyond Fick's law of diffusion? Biochim Biophys Acta 1778: 2154–2156. 18510944 10.1016/j.bbamem.2008.05.001 PMC3045803

[28] Martinac B, Hamill OP (2002) Gramicidin A channels switch between stretch activation and stretch inactivation depending on bilayer thickness. Proc Natl Acad Sci U S A 99: 4308–4312. 11904391 10.1073/pnas.072632899 PMC123644

[29] Tian A, Johnson C, Wang W, Baumgart T (2007) Line tension at fluid membrane domain boundaries measured by micropipette aspiration. Phys Rev Lett 98: 208102. 17677743 10.1103/PhysRevLett.98.208102

[30] Woolf TB, Roux B (1996) Structure, energetics, and dynamics of lipid-protein interactions: A molecular dynamics study of the gramicidin A channel in a DMPC bilayer. Proteins 24: 92–114. 8628736 10.1002/(SICI)1097-0134(199601)24:1<92::AID-PROT7>3.0.CO;2-Q

[31] Kota Z, Pali T, Marsh D (2004) Orientation and Lipid-Peptide Interactions of Gramicidin A in Lipid Membranes: Polarized Attenuated Total Reflection Infrared Spectroscopy and Spin-Label Electron Spin Resonance. Biophys J 86: 1521–1531. 14990479 10.1016/S0006-3495(04)74220-4 PMC1303987

[32] Nielsen C, Goulian M, Andersen OS (1998) Energetics of inclusion-induced bilayer deformations. Biophys J 74: 1966–1983. 9545056 10.1016/S0006-3495(98)77904-4 PMC1299538

[33] Lundbaek JA, Collingwood SA, Ingolfsson HI, Kapoor R, Andersen OS (2010) Lipid bilayer regulation of membrane protein function: gramicidin channels as molecular force probes. J R Soc Interface 7: 373–395. 19940001 10.1098/rsif.2009.0443 PMC2842803

[34] Rokitskaya TI, Kotova EA, Antonenko YN (2003) Tandem Gramicidin Channels Cross-linked by Streptavidin. J Gen Physiol 121: 463. 12719486 10.1085/jgp.200208780 PMC2217381

[35] Goforth RL, Chi AK, Greathouse DV, Providence LL, Koeppe REII, et al (2003) Hydrophobic Coupling of Lipid Bilayer Energetics to Channel Function. J Gen Physiol 121: 477. 12719487 10.1085/jgp.200308797 PMC2217378

[36] Al-Momani L, Reiss P, Koert U (2005) A lipid dependence in the formation of twin ion channels. Biochem Biophys Res Commun 328: 342–347. 15670789 10.1016/j.bbrc.2004.12.170

[37] Krylov AV, Kotova EA, Yaroslavov AA, Antonenko YN (2000) Stabilization of O-pyromellitylgramicidin channels in bilayer lipid membranes through electrostatic interaction with polylysines of different chain lengths. Biochim Biophys Acta 1509: 373–384. 11118547 10.1016/s0005-2736(00)00320-5

[38] Saffman PG, Delbruck M (1975) Brownian motion in biological membranes. Proc Natl Acad Sci U S A 72: 3111–3113. 1059096 10.1073/pnas.72.8.3111 PMC432930

[39] Sackmann E, Merkel R (2012) Lehrbuch der Biophysik. Weinheim: Wiley-VHC Verlag & Co. KGaA.

[40] Maier B, Radler JO (1999) Conformation and self-diffusion of single DNA molecules confined to two dimensions. Phys Rev Lett 82: 1911–1914.

[41] Milligan G (2004) G Protein-Coupled Receptor Dimerization: Function and Ligand Pharmacology. Molecular Pharmacology 66: 1–7. 15213289 10.1124/mol.104.000497.

[42] Cornea A, Janovick JA, Maya-Nunez G, Conn PM (2001) Gonadotropin-releasing Hormone Receptor Microaggregation. J Biol Chem 276: 2153–2158. 11035030 10.1074/jbc.M007850200

[43] Hawtin SR, Simms J, Conner M, Lawson Z, Parslow RA, et al (2006) Charged Extracellular Residues, Conserved throughout a G-protein-coupled Receptor Family, Are Required for Ligand Binding, Receptor Activation, and Cell-surface Expression. J Biol Chem 281: 38478–38488. 16990262 10.1074/jbc.M607639200

[44] Wess J (1997) G-protein-coupled receptors: molecular mechanisms involved in receptor activation and selectivity of G-protein recognition. FASEB J 11: 346–354. 9141501

